# Influenza Amongst Horses

**Published:** 1845-05

**Authors:** 


*Influenza amongst Horses.*—This epidemic has been for the last two weeks very prevalent amongst horses. The leading symptoms of the disorder are swelling and inflammation of the throat, discharge from the mucous membrane of the nose, and lassitude and want of strength. Messrs. Pickford, the celebrated carriers, have upwards of 100 horses now suffering from the complaint, and it is very prevalent and fatal amongst dray horses, one brewer at Westminster having lost three out of eighteen that were attacked. The Paddington Omnibus Conveyance Company have been great sufferers, having lost nearly fifty horses within the last month, and had no less than 105 laid up at one time.—*London Times.*

